# Internal parasites of the Eurasian beaver (*Castor fiber*) in Lesser Poland region (Poland) - occurrence and diversity

**DOI:** 10.1016/j.ijppaw.2026.101305

**Published:** 2026-10-16

**Authors:** Marta Basiaga, Adam Wójcicki, Jerzy Kowal, Marek Wajdzik

**Affiliations:** a University of Agriculture in Kraków, Faculty of Animal Science, Department of Zoology and Animal Welfare, Al. Mickiewicza 24/28, Kraków, 30-059, Poland; b Forest Research Institute, Department of Forest Ecology, ul. Braci Leśnej 3, Sękocin Stary, Raszyn, 05-090, Poland; c University of Agriculture in Kraków, Faculty of Forestry, Department of Forest Biodiversity, Al. 29 Listopada 46, Kraków, 31-425, Poland

**Keywords:** *Travassosius rufus*, *Stichorchis subtriquetrus*, Parasite burden, Wildlife, Rodents

## Abstract

Between 2017 and 2019, necropsies were performed on 26 Eurasian beavers (*Castor fiber*) from the Lesser Poland Voivodeship (Małopolska Region), Poland, to investigate the composition of their helminth fauna. Two host-specific helminth species were identified - *Stichorchis subtriquetrus* and *Travassosius rufus*. Trematode *S. subtriquetrus* was more prevalent, infecting 88.5% of beavers (95% CI: 69.8-97.6%) with a mean intensity of 132.7 ± 114.7 parasites per host (range: 2-454), whereas nematode *T. rufus* occurred in 69.2% of hosts (95% CI: 48.2-85.7%), with a mean intensity of 306.7 ± 493.3 (range: 7-2057). The abundance of neither *S. subtriquetrus* nor *T. rufus* was significantly associated with host sex, body mass, age or landscape type, season, and site (p > 0.05). Likewise, no significant correlation was found between the abundances of the two helminth species (p > 0.05). In contrast, the numbers of male and female *T. rufus* recovered from individual hosts were strongly positively correlated (r = 0.98, p < 0.001), with females consistently predominating. These findings are consistent with previous studies reporting the dominance of these species in beavers.

## Highlights

• Two helminths were identified, i.e. *Stichorchis subtriquetrus* and *Travassosius rufus*.
• All examined beavers harboured at least one host-specific helminth species.
• Mixed infections occurred in more than half of the examined beavers.
• Helminth abundance was not associated with host or environmental factors.

## Graphical abstract

**Figure:**
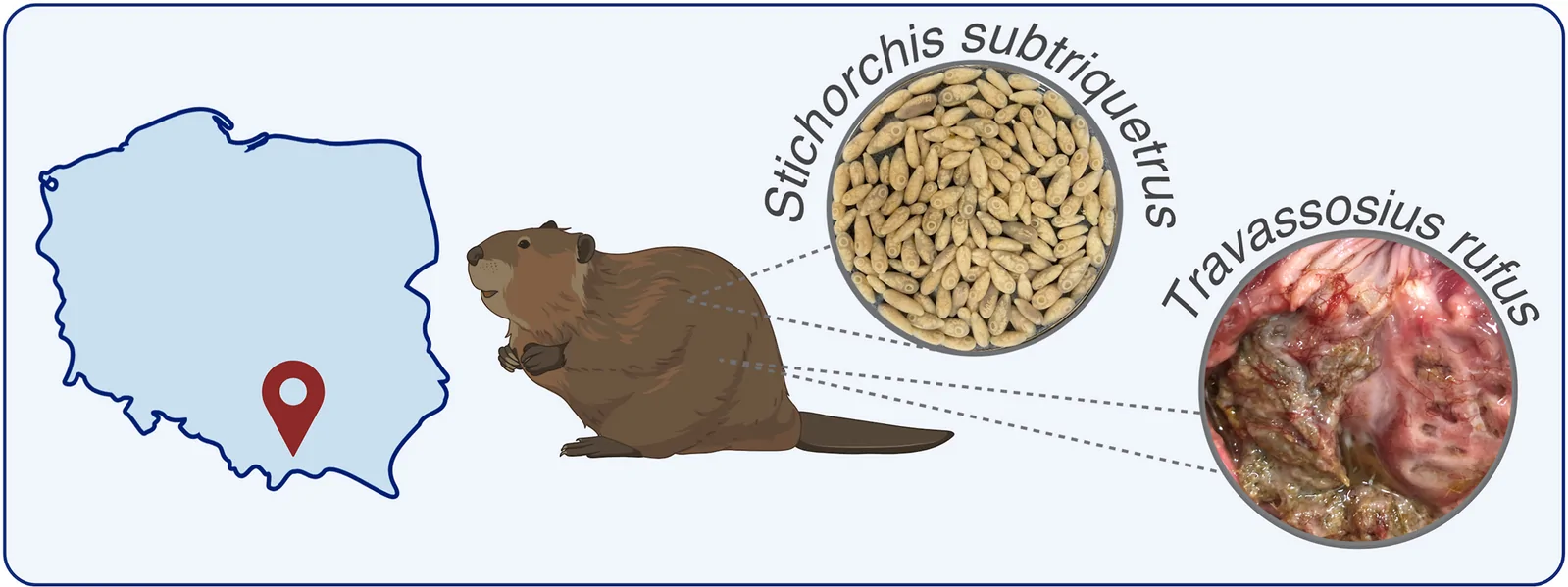

## 1 Introduction

The Eurasian beaver (*Castor fiber*) is the largest semi-aquatic rodent (Rodentia: Castoridae) in Europe. According to Paderewski and Sienkiewicz-Paderewska (2025), the Polish beaver population increased at an average annual rate of nearly 11% between 1960 and 2023, rising from 217 to 154,431 individuals. Outside Poland, populations exceeding 100,000 individuals have been reported in Latvia, Lithuania and Sweden, whereas the largest population of Eurasian beaver occurs in Russia (Halley et al., 2020). Substantial populations are also present in several other European countries (Halley et al., 2020; Wróbel, 2020). Consequently, the conservation status of the Eurasian beaver on the IUCN Red List was changed from Near Threatened to Least Concern (Batbold et al., 2021).

Despite this favourable conservation status, the Eurasian beaver remains protected under national legislation in many European countries. Within the European Union, the species is listed in Annexes II, IV and/or V of the Habitats Directive (Council Directive 92/43/EEC), requiring Member States to designate special areas of conservation and to ensure strict protection of the species. However, under Article 16 of the Directive, derogations, including culling and hunting, may be authorised. Carcasses obtained through these practices, as well as those of animals found dead in the wild, constitute valuable material for research on Eurasian beaver biology, ecology, and health, including parasitological investigations.

Following both illegal releases and legal reintroduction of the Eurasian beaver into numerous European countries (Bertolino et al., 2023), where populations had declined substantially or the species had been locally extirpated (Kitchener and Conroy, 1997; Nolet and Rosell, 1998; Halley et al., 2012, 2020), there is an increasing need to understand which infectious agents may be introduced with this host. This is particularly important because some of these pathogens may pose a threat to wildlife, domestic animals and humans. In addition to recent reports documenting bacterial and viral infections in Eurasian beavers (Monecke et al., 2021; Maas et al., 2022; Ellenberger et al., 2024; Surján et al., 2024; Sedlák et al., 2025; White et al., 2025), attention has also been paid to parasitic infections affecting this species. Studies addressing the parasitological implications of beaver reintroductions and their presence in the environment from a One Health perspective have focused predominantly on protozoan parasites. These include *Toxoplasma gondii*, reported in Germany (Herrmann et al., 2013), the Netherlands (Maas et al., 2022), Switzerland (Scherrer et al., 2024), England (Jacquart et al., 2025), and the Czech Republic (Sedlák et al., 2025); *Giardia* spp. – in Poland (Bajer et al., 2008), Italy (Turillazzi et al., 2024) and Scotland (Pizzi et al., 2026); *Cryptosporidium* spp. – in Poland (Bajer et al., 2008), Great Britain (Campbell-Palmer et al., 2021) and Slovakia (Šmigová et al., 2025), and *Blastocystis* spp., reported in Slovakia (Šmigová et al., 2025). Furthermore, Sedlák et al. (2025) reported the occurrence of *Neospora caninum* in *C. fiber* from the Czech Republic.

Metazoan parasites commonly reported in Eurasian beavers are generally not considered to have substantial effects on wildlife health or ecosystem functioning. Nevertheless, the confirmed occurrence of the zoonotic cestode *Echinococcus multilocularis* in this host warrants attention. The first case was reported in Switzerland by Janovsky et al. (2002), followed by subsequent records from the United Kingdom (Barlow et al., 2011), Serbia (Ćirović et al., 2012), and Austria (Posautz et al., 2015). Notably, the cases from Serbia and the United Kingdom represented the first records of *E. multilocularis* in free-ranging wildlife in these countries, and in both instances the infected beavers had been translocated from Bavaria, Germany, which is recognised as an endemic area for *E. multilocularis* (EFSA et al., 2025). In addition, infection with *E*. *granulosus* has also been confirmed in the Eurasian beaver. Romaschov (1969) reported the occurrence of this tapeworm in beavers in Russia. Subsequently, Tkach et al. (2002) molecularly confirmed the occurrence of *E. granulosus* in a beaver from Poland. A more comprehensive parasitological analysis of the same group of beavers was later published by Dróżdż et al. (2004), and the species was recorded again by Demiaszkiewicz et al. (2014). Although beavers do not constitute a major prey species for one of the definitive hosts of *Echinococcus* spp., the gray wolf (*Canis lupus*), they may form part of its diet in areas where both species coexist (Mysłajek et al., 2021). Beaver remains have also been reported, albeit only occasionally, in the diet of the red fox (*Vulpes vulpes*) (Kidawa and Kowalczyk, 2011). This is consistent with the findings of Kosmider et al. (2013), who assessed the risk of introducing *E. multilocularis* into the United Kingdom through beaver reintroductions and concluded that the risk is substantial when infected animals are released into environments inhabited by definitive hosts, particularly wild canids capable of preying on or scavenging beavers.

Among zoonotic parasites, nematodes of the genus *Trichinella* also pose a potential threat to public health (Malone et al., 2024). To date, *T*. *spiralis* has been reported in Eurasian beavers in Poland (Różycki et al., 2020), whereas *T*. *britovi* has been detected in Latvia (Bērziņa et al., 2012; Segliņa et al., 2015). Although beaver meat consumption remains limited in Europe, increasing beaver populations and the implementation of population measures, including culling and hunting in several countries (Iso-Touru et al., 2020), may contribute to its wider use as a food source. Importantly, Bronstein and Lukashev (2018) described a human case of trichinellosis in Russia and suggested that the infection may have been associated with the consumption of beaver meat.

The aforementioned reports highlight the potential public health risks associated with the consumption of beaver meat and exposure to areas contaminated with beaver faeces. They also underscore the importance of monitoring infectious agents in beaver populations. Therefore, both reintroduction and translocation programmes involving *C. fiber*, as well as the management of areas where this rodent occurs, should consider the potential introduction and spread of parasites, including zoonotic species.

The helminth fauna of the Eurasian beaver is dominated by host-specific parasites, particularly the trematode *Stichorchis subtriquetrus* (Rudolphi, 1814) and the nematode *Travassosius rufus* (Khalil, 1922). Nevertheless, several additional helminth species have also been documented. Table 1 compiles data from studies published between 2000 and 2025 on helminth species reported in *C. fiber*, including information on their geographic distribution, detection methods, and infection parameters. The most relevant findings are discussed further in the following section. These studies documented 13 helminth species, comprising three trematodes, three cestodes, and seven nematodes. In contrast, Romaschov (1969) recognised 28 helminth species in his review, whereas Romashov (2012) reported 24 species in this host. Given the continued detection of previously unreported parasite species in Eurasian beavers (e.g. *Trichinella* spp.), alongside the apparent disappearance of others reported previously, further research is needed to improve our understanding of the parasite fauna of this host. Based on the literature review and the increasing size and density of Eurasian beaver populations, we expected host-specific helminth species to predominate, while additional helminth taxa, including species of potential veterinary or public health importance, might also be noted.

**Table 1:** Overview of published studies on helminths of Eurasian beavers (*Castor fiber*) (2000-2025).

Species	Country	Detection method	Infection parameters^a^	Reference
***Stichorchis subtriquetrus***	Belarus	N	4/4	Strużyński et al. (2016)
	Croatia	N	1/1	Sager et al. (2005)
	Czech Republic	N, FA	9/11 (82%)	Máca et al. (2015)
		N, MA	14/15 (93%)	Benovics et al. (2022)
	Hungary	N, FA, MA	N - 38/47 (80.9%); FA - 15/47 (32%)	Szekeres et al. (2022)
	Lithuania	N	1/2	Mažeika et al. (2003)
	Poland	N, FA	N - 40/43 (93%), FA - 70/136 (51%)	Dróżdż et al. (2004)
		N, FA	N - 45/48 (93.7%) FA - 40/48 (83.3%)	Demiaszkiewicz et al. (2014)
		N	9/10 (90%)	Demkowska-Kutrzepa et al. (2016)
		N	23/26 (88.5%)	present study
	Russia	N	24/25 (96%)	Andreyanov et al. (2021)
	Scotland	N, MA	1/1	Campbell-Palmer et al. (2013)
	Serbia	N	1/8 (12.5%)	Ćirović et al. (2009)
	Slovakia	N	1/1	Koubková et al. (2002)
		FA	5/16 (31.3%)	Bystrianska et al. (2021)
		N, FA	1/1	Lazár et al. (2023)
	Slovenia	N	2/2	Vengušt et al. (2009)
	Sweden	N	30/30	Åhlen et al. (2021)
	United Kingdom	N, FA	N - 4/30 (13.3%), FA - 16/84 (19%)	Campbell-Palmer et al. (2021)
***Travassosius rufus***	China	N, MA	1/1	Jia et al. (2025)
	Czech Republic	N, MA	1/15 (6.7%)	Benovics et al. (2022)
	Italy	FA, MA	2/2	Turillazzi et al. (2024)
	Lithuania	N	2/2	Mažeika et al. (2003)
	Poland	N, FA	N - 27/43 (63%), FA - 19/136 (14%)	Dróżdż et al. (2004)
		N, FA	N - 33/48 (68.7%) FA - 29/48 (60.4%)	Demiaszkiewicz et al. (2014)
		N	18/26 (69.2%)	present study
	Russia	N	22/25 (88%)	Andreyanov et al. (2021)
	Slovakia	FA	4/18 (22.2%)	Bystrianska et al. (2021)
	Sweden	N	30/30	Åhlen et al. (2021)
***Stichorchis subtriquetrus* and/or *Travassosius rufus***	Norway	N, FA	8/29 (27.6%)	Goodman et al. (2012)
	Scotland	nd	6/16 (37.5%)	Goodman et al. (2017)
***Trichinella britovi***	Latvia	AD, MA	1/65 (1.5%)	Bērziņa et al. (2012)
		AD, MA	1/182 (0.5%)	Segliņa et al. (2015)
***Trichinella spiralis***	Poland	AD, MA	1/69 (1.4%)	Różycki et al. (2020)
***Echinococcus granulosus***	Poland	N	1/43 (2.3%)	Dróżdż et al. (2004)
		N	1/48 (2%)	Demiaszkiewicz et al. (2014)
***Echinococcus multilocularis***	Austria	N, MA	6/398 (1.5%)	Posautz et al. (2015)
	Germany	N, IT, MA	1/45 (2.2%)	Campbell-Palmer et al. (2015)
	Serbia	N	1/1	Ćirović et al. (2012)
	Switzerland	N	1/1	Janovsky et al. (2002)
	United Kingdom	N, MA	1/1	Barlow et al. (2011)
***Taenia martis***	Germany	N, IT, MA	1/45 (2.2%)	Campbell-Palmer et al. (2015)
***Psilotrematoides castoris***	Poland	N	4/48 (8.3%)	Demiaszkiewicz et al. (2014)
***Fasciola hepatica***	Belarus	N	2/20 (10%)	Shimalov and Shimalov (2000)
***Calodium hepaticum***	Czech Republic	N, MA	1/15 (6.7%)	Benovics et al. (2022)
***Trichostrongylus axei***	Poland	N	6/43 (14%)	Dróżdż et al. (2004)
***Trichostrongylus capricola***	Poland	N	1/48 (2%)	Demiaszkiewicz et al. (2014)
***Trichuris* spp.**	Italy	FA, MA	2/2	Turillazzi et al. (2024)

Abbreviations: AD - artificial digestion method; FA - fecal analysis; IT - diagnostic imaging techniques; MA - molecular analysis; N – necropsy; SM - serological methods; nd - no data.

a Infected/examined (prevalence).

The aim of this study was to assess the helminth fauna and infection parameters of Eurasian beavers culled as part of a population control programme in the Lesser Poland Voivodeship, southern Poland.

## 2 Materials and methods

### 2.1 Origin of beavers and post-mortem examination

The study material consisted of 26 Eurasian beaver carcasses obtained from the wild during the 2017/2018 and 2018/2019 hunting seasons in six hunting districts located in the Lesser Poland Voivodeship (southern Poland). Detailed data on the examined animals are provided in Appendix A, Table A.1. In Poland, *C. fiber* is subject to partial species protection under national legislation, with the taking of individuals from the wild potentially permitted as a population management measure, in accordance with Council Directive 92/43/EEC (Annexes II and V). The beavers were culled as part of population management measures under an agreement between the General Directorate for Environmental Protection and the Polish Hunting Association, concluded on 19 July 2016, and based on permits issued by the Regional Directorate for Environmental Protection in Kraków to the lessees or managers of hunting districts.

Cadavers were dissected following standard parasitological procedures, with attention to the recovery of parasites from the organs and body cavities. The gastrointestinal tract was examined by separating it into the following sections: oesophagus, stomach, small intestine, caecum, and the remaining large intestine. Each section was opened longitudinally, and the mucosal surface was scraped. The intestinal contents, together with the mucosa, were washed through a 250 μm mesh sieve using tap water, and the retained material was transferred in portions to Petri dishes. The liver and lungs were dissected along the major and smaller ducts and cut into smaller pieces. The dissected lung tissue was then placed in a beaker containing physiological saline and manually compressed, after which the sediment was allowed to settle and subsequently transferred to Petri dishes. The liver, spleen, and kidneys were examined by placing small tissue fragments between two glass plates and gently compressing them. All recovered materials, as well as the heart, trachea, gallbladder, and urinary bladder, were examined for parasites under a stereomicroscope. Recovered helminths were rinsed in water or physiological saline, fixed in 70% ethanol, and identified morphologically. Trematodes were stained with alcoholic borax carmine, whereas nematodes were cleared in lactophenol. Species identification was based on standard diagnostic morphological features and relevant morphometric measurements, following the descriptions of Weidman (1918), Khalil (1922), and Koubková et al. (2002).

Parasitological parameters of helminth infection in beavers were analysed in relation to individual (sex, age, and body mass) and environmental factors (season, landscape type, and site).

### 2.2 Statistical analysis

All statistical analyses were performed using Statistica version 14.1.0.4 (TIBCO Software Inc.). The study included 26 beavers sampled at six study sites, comprising 16 males and 10 females. Parasite abundance was defined as the number of individuals of a given parasite species recorded in each beaver. The abundances of *S*. *subtriquetrus* and *T*. *rufus* were analysed separately. Differences in parasite abundance between male and female beavers, between young and adult beavers, and between forest and agricultural landscapes were assessed using the Mann–Whitney *U* test. Beavers were classified as young (1–2.5 years) or adults (≥3 years). Hunting districts with at least 40% forest cover were classified as forest landscapes, whereas those with less than 40% forest cover were classified as agricultural landscapes. Differences in parasite abundance among the six study sites were assessed using the Kruskal–Wallis test. This test was used to determine whether the abundance of each parasite species differed among sampling sites. The relationships between parasite abundance and host body mass were assessed using Spearman's rank correlation coefficient. Spearman's rank correlation coefficient was also used to evaluate the association between the abundances of *S. subtriquetrus* and *T. rufus*, as well as to assess the co-abundance of *T. rufus* males and females within individual beavers. All statistical tests were two-tailed, and statistical significance was set at p < 0.05.

## 3 Results

Two helminth species, *S*. *subtriquetrus* and *T*. *rufus,* were identified in the examined beavers. All individuals were infected with at least one helminth species, while mixed infections were recorded in 15 beavers (57.6%). *S. subtriquetrus* was detected in 23 individuals, with a prevalence of 88.5% (95% CI: 69.8-97.6%). The mean intensity of infection was 132.7 ± 114.7 parasites (range: 2-454). *T. rufus* was detected in 18 beavers, with a prevalence of 69.2% (95% CI: 48.2-85.7%) and a mean intensity of 306.7 ± 493.3 parasites (range: 7-2057). Detailed results of parasite infections in Eurasian beavers, including the effects of host-related and environmental factors, are presented in Table 2. *T. rufus* specimens were recovered predominantly from the stomachs of the examined beavers, with only a few individuals found in the small intestine, whereas most *S. subtriquetrus* specimens were recovered from the caecum, followed by the remaining sections of the large intestine, with only a few individuals found in the small intestine.

**Table 2:** Parasitological parameters of helminth infections in Eurasian beavers in relation to individual and environmental factors.

	S. subtriquetrus				*T. rufus*			
	N_i_/N	I	SD	range	Ni/N	I	SD	range
**season**								
2017/2018	9/9	136.6	102.6	2-264	6/9	203.3	206.2	22-494
2018/2019	14/17	130.3	125.6	10-454	12/17	358.3	589.9	7-2057
**sex**								
female	8/10	166.3	146.5	10-454	7/10	159.1	163.6	7-378
male	15/16	114.9	94.6	2-264	11/16	400.5	610.4	7-2057
**age**								
young (1-2.5)	8/9	131.8	97.5	10-264	7/9	561	760.6	7-2057
adult (≥3)	15/17	133.3	126.2	2-454	12/17	179.5	245.3	7-868
**landscape**								
forest	11/12	162.5	129.9	2-454	8/12	106.3	137.1	22-402
agricultural	12/14	105.5	96.3	7-264	10/14	467	617	7-2057

Ni - number of infected individuals, N - number of examined individuals, I - mean intensity of infection, SD – standard deviation.

The Mann–Whitney *U* test analysis showed that the abundance of *S. subtriquetrus* and *T. rufus* was not statistically significantly correlated with host sex, age group or landscape type (p > 0.05; Table 3). The abundance of *S. subtriquetrus* and *T. rufus* was also not correlated with study sites (p > 0.05; Table 4). Likewise, the abundance of neither parasite species was significantly correlated with host body mass (p > 0.05; Table 5). In addition, the abundances of *S. subtriquetrus* and *T. rufus* were not correlated (p > 0.05; Table 5). By contrast, the numbers of male and female *T. rufus* recovered from individual hosts were strongly positively correlated, with females consistently outnumbering males (r = 0.98, p < 0.001; Table 5). Overall, 5520 *T. rufus* specimens were collected, comprising 3084 females (56%) and 2436 males (44%).

**Table 3:** The Mann-Whitney *U* test results for *S. subtriquetrus* and *T. rufus* by beaver sex, age group, landscape, and site.

Variables correlation	U	Z	p
***S. subtriquetrus* & sex**	79.00	−0.03	0.98
***S. subtriquetrus* & age group**	74.00	0.11	0.92
***S. subtriquetrus* & landscape**	66.00	−1.00	0.32
***T. rufus* & sex**	65.50	−0.50	0.60
***T. rufus* & age group**	53.50	0.82	0.41
***T. rufus* & landscape**	53.00	1.33	0.19

U – U value, Z – z-value, p – p-value (2-tailed).

**Table 4:** The Kruskall-Wallis test results for *S. subtriquetrus* and *T. rufus* and study sites.

	H	χ^2^	df	p
***S. subtriquetrus* & study site**	71.50	5.20	5	0.39
***T. rufus* & study site**	37.00	4.57	5	0.47

H – H statistic, χ^2^ - chi-square, df – degrees of freedom, p – p-value.

**Table 5:** Spearman's correlation coefficients for the co-abundance of *S. subtriquetrus* and *T. rufus*, for their abundance in relation to beaver body mass, and for the co-abundance of *T. rufus* males and females.

Variables correlation	R	t(N-2)	p
***S. subtriquetrus* & body mass**	0.16	0.81	0.43
***S. subtriquetrus* & *T. rufus***	0.19	0.95	0.35
***T. rufus* & body mass**	−0.18	−0.89	0.38
***T. rufus* males *& T. rufus* females**	0.99	31.38	< 0.001*

R - correlation coefficient, t(N-2) - t-distribution with (N-2) degrees of freedom, p - p-value.

## 4 Discussion

*S. subtriquetrus* and *T. rufus* are the characteristic and most frequently reported helminths of the Eurasian beaver (see Table 1), with prevalence ranges of 12.5-100% and 6.7-100%, respectively. In Poland, the first survey of beaver parasites was conducted by Joszt (1964), who reported *Ascaris castoris*, *Trichostrongylus capricola* and *S. subtriquetrus*. Subsequent studies by Dróżdż et al. (2000) expanded the known helminth fauna by reporting *T. rufus* for the first time, while Tkach et al. (2002) added *E. granulosus* to the list. Later investigations (Dróżdż et al., 2004; Demiaszkiewicz et al., 2014; Demkowska-Kutrzepa et al., 2016; Różycki et al., 2020) documented the occurrence of *Psilotrematoides castoris*, *Trichostrongylus axei, T. capricola*, and *T. spiralis*. Despite the growth of the Eurasian beaver population in Poland (Paderewski and Sienkiewicz-Paderewska, 2025), information on its health status and infectious diseases remains limited. Nevertheless, studies of the gastrointestinal helminth community have consistently shown that the fauna is dominated by two host-specific species.

*S. subtriquetrus* is a large trematode with a body that tapers anteriorly and bears two well-developed suckers. Depending on the intensity of infection, stichorchiosis may range from a subclinical condition to an acute, potentially fatal form (Romaschov, 1969; Donald et al., 2021). In our study, the mean infection intensity was 132.7 ± 114.7 specimens per host, with 12 of the 23 infected beavers harbouring more than 100 flukes, and a maximum burden of 454 individuals. However, substantially higher infection intensities have been reported, reaching 893 (Demiaszkiewicz et al., 2014), 736 (Dróżdż et al., 2004), and 652 (Strużyński et al., 2016). Likewise, the prevalence of *S. subtriquetrus* is consistently high, with post-mortem surveys typically reporting values between 80% and 93% (Table 1); in the present study, prevalence was 88.5%. Although several studies have reported 100% prevalence, these findings were generally based on examinations of a limited number of beavers (n ≤ 4). An exception is the study by Åhlen et al. (2021), in which the fluke was detected in all 30 examined animals.

The potential clinical significance of high parasite burdens is supported by reports of pathological changes associated with *S. subtriquetrus,* including inflammatory lesions in the caecum (Demiaszkiewicz et al., 2014). Similarly, Niemiec et al. (2016) described chronic inflammation of the small intestine, characterised by thickened villi, along with lymphocytic and eosinophilic infiltrates associated with *S. subtriquetrus*. Although the caecum is the primary site of infection, the fluke has occasionally been reported in the small intestine, a finding that was also confirmed in the present study. Notably, Demiaszkiewicz et al. (2014) and Åhlen et al. (2021) reported only juvenile specimens in this intestinal section. Moreover, Donald et al. (2021) described three cases of captive Eurasian beavers that died in association with *S. subtriquetrus* infection. In one individual, focal ulceration and haemorrhage of the large intestine, together with poor body condition, were attributed to a heavy parasite burden, while translocation-related stress was suggested as a factor in clinical stichorchiosis. This is consistent with Northover et al. (2018), who noted that translocation may compromise host immunocompetence, increasing susceptibility to parasitic infections and exacerbating the pathological effects of pre-existing parasite burdens. Therefore, parasitic infections should be carefully considered during beaver translocations, and appropriate parasite screening, surveillance, and control programmes should be incorporated into translocation protocols. Furthermore, from an epizootiological and One Health perspective, translocated beavers may introduce parasites into new areas, including species that pose a risk to wildlife, domestic animals, or humans (see Introduction). In the first report of *S. subtriquetrus* in the Czech Republic, Koubková et al. (2002) proposed that the parasite had been introduced with Eurasian beavers translocated from Poland. Similar patterns have been documented in Serbia (Ćirović et al., 2009), Croatia (Sager et al., 2005), Slovenia (Vengušt et al., 2009), and Scotland (Campbell-Palmer et al., 2013), where *S. subtriquetrus* was first detected shortly after the introduction of imported beavers, promoting the colonisation of new areas by the parasite.

Another core component of the beaver parasite community is the stomach nematode *T. rufus*. However, despite its frequent occurrence, information on the pathological effects of infection remains scarce. Demiaszkiewicz et al. (2014) reported that heavy infections were associated with inflammatory lesions of the gastric mucosa. In their study, *T. rufus* was detected in 33 of 48 examined beavers (68.7%), with a mean intensity of 818 parasites per host (range: 10-4336). Nevertheless, even higher parasite burdens have been documented. Jia et al. (2025) reported a single beaver harbouring 5478 nematodes. In the present study, the mean intensity of *T. rufus* infection was 306.7 ± 493.3 parasites, with a maximum intensity of 2057 individuals. The prevalence in our study (69.2%) was comparable to previous studies conducted in Poland (Dróżdż et al., 2004; Demiaszkiewicz et al., 2014), but lower than that reported in Sweden (100%; Åhlen et al., 2021) and Russia (88%; Andreyanov et al., 2021).

In the present study, endoparasite infection parameters in Eurasian beavers were not significantly affected by individual or environmental factors. Similarly, Máca et al. (2015) found no association between *S. subtriquetrus* infection and host age or sex in the Czech Republic. In contrast, Demiaszkiewicz et al. (2014) reported age-related differences in the infection parameters of *T. rufus* and *S. subtriquetrus*. Compared with adults, younger beavers (≤2 years) showed lower prevalence of both parasite species. However, the mean intensity of *S. subtriquetrus* infection was higher in young animals, whereas no such pattern was observed for *T. rufus*. It should be noted, however, that the age groups differed considerably in sample size. The same authors also reported seasonal variation in infection parameters. For *S. subtriquetrus*, prevalence ranged from 87.5% to 100%, whereas mean intensity varied between 51 and 222 parasites per host, with the lowest values recorded in summer and the highest in autumn. In contrast, *T. rufus* showed the lowest infection parameters in autumn and the highest in spring. Dróżdż et al. (2004) found no age-related differences in *S. subtriquetrus* infection, with prevalence reaching 89% in first-year beavers and 96% in adults. In contrast, marked differences were observed for *T. rufus,* with prevalence of 39% in young beavers and 80% in adults. Bystrianska et al. (2021) reported interannual variation in the infection parameters of both *T. rufus* and *S. subtriquetrus* in beavers from Slovakia. However, unlike the aforementioned studies, their results were based exclusively on coprological examinations. Åhlen et al. (2021) also highlighted the importance of host age as well as environmental conditions in shaping parasite infections. Beavers from southern Sweden, where temperatures are generally higher, had more newly acquired trematode infections and higher intensities of adult nematodes than individuals from northern regions. In addition, one-year-old beavers harboured heavier *S. subtriquetrus* infections than older animals, whereas no age-related differences were observed for *T. rufus*. Furthermore, Åhlen et al. (2021) suggested that the 100% prevalence of endoparasites in Swedish beavers may be associated with the low genetic diversity of the host population, which could limit the functional capacity of the immune system and consequently increase susceptibility to parasitic infections.

## 5 Conclusions

Our study confirms that the gastrointestinal helminth community of the Eurasian beaver in Poland is consistently dominated by the host-specific species *Stichorchis subtriquetrus* and *Travassosius rufus*, with prevalence and infection intensity comparable to those reported in previous post-mortem surveys. Neither host-related factors (body mass, age, or sex) nor landscape type significantly influenced infection parameters, suggesting that these parasites are widespread and well established in the studied beaver population. Given the high prevalence of these parasites and the documented pathological effects associated with heavy infections, parasitological surveillance should be considered an integral component of Eurasian beaver management, particularly in translocation programmes. Such monitoring is important not only for minimising potential health impacts on translocated animals but also for assessing the risk of parasite introduction into newly colonised areas. At the same time, the conservation value of host-specific parasite fauna should be recognised, highlighting the need to balance disease management with broader biodiversity conservation objectives.

## CRediT authorship contribution statement

**Marta Basiaga:** Writing – review & editing, Writing – original draft, Visualization, Methodology, Investigation, Conceptualization. **Adam Wójcicki:** Writing – review & editing, Visualization, Formal analysis. **Jerzy Kowal:** Writing – review & editing, Investigation. **Marek Wajdzik:** Writing – review & editing, Resources.

## Funding

This work was supported by the 10.13039/501100004569Ministry of Science and Higher Education (Poland) (subvention number: 020014-DO15).

## Declaration of competing interests

The authors declare no conflict of interest.

## Figures and Tables

**Table A.1:** Individual data and parasite infection results for the examined Eurasian beavers

No.	Season	Age [years]	Sex	Body mass [kg]	Color morph	Hunting district	*Stichorchis subtriquetrus*	*Travassosius rufus*	Female *T. rufus*	Male *T. rufus*
**1**	2017/2018	1	male	7	black	79⁎	207	28	16	12
**2**	2017/2018	1	male	9	brown	78⁎	51	402	206	196
**3**	2017/2018	2,5	male	14	black	93	264	494	271	223
**4**	2017/2018	3	male	16	brown	79⁎	2	47	17	30
**5**	2017/2018	>3	female	25	brown	79⁎	232	22	14	8
**6**	2017/2018	>3	female	26	gray	79⁎	211	227	119	108
**7**	2017/2018	>3	male	28	black	79⁎	182	-	-	-
**8**	2017/2018	>3	male	22	black	125	7	-	-	-
**9**	2017/2018	>3	male	22	brown	78⁎	73	-	-	-
**10**	2018/2019	1	female	9	black	95	10	-	-	-
**11**	2018/2019	1	female	9	black	95	14	-	-	-
**12**	2018/2019	1	female	9	brown	94	216	7	3	4
**13**	2018/2019	2	female	13	black	94	-	378	190	188
**14**	2018/2019	2	male	13	black	95	164	-	-	-
**15**	2018/2019	2,5	male	15	brown	95	128	2057	1104	953
**16**	2018/2019	3	female	21	black	95	-	57	36	21
**17**	2018/2019	3	female	20	black	95	70	370	244	126
**18**	2018/2019	3	male	21	black	95	56	7	6	1
**19**	2018/2019	3	male	18	brown	95	49	247	132	115
**20**	2018/2019	3	male	20	brown	79⁎	17	-	-	-
**21**	2018/2019	3	male	18	gray	95	253	868	560	308
**22**	2018/2019	>3	female	24	brown	79⁎	123	-	-	-
**23**	2018/2019	>3	female	26	brown	79⁎	454	53	21	32
**24**	2018/2019	>3	male	26	black	79⁎	-	34	18	16
**25**	2018/2019	>3	male	24	black	95	35	185	105	80
**26**	2018/2019	>3	male	26	brown	79⁎	235	37	22	15

⁎ forest hunting districts.
